# Supplementary material for: Early activation of cellular stress and death pathways caused by cytoplasmic TDP-43 in the rNLS8 mouse model of ALS and FTD
Source: Mol Psychiatry. 2023 Apr 3;28(6):2445–61. doi: 10.1038/s41380-023-02036-9 (PMC10611572; doi:10.1038/s41380-023-02036-9)
Supplement: Supplementary file 1 — Supp figures [file 41380_2023_2036_MOESM1_ESM.pdf]

Supplementary materials

Title: Early activation of cellular stress and death pathways caused by cytoplasmic TDP-43 in the rNLS8 mouse model of ALS and FTD

Authors:

Wei Luan[1], Amanda L. Wright[1,2], Heledd Brown-Wright[1], Sheng Le[2], Rebecca San Gil[1], Lidia Madrid San Martin[1], Karen Ling[3], Paymaan Jafar-Nejad[3], Frank Rigo[3], Adam K. Walker[1,2,*]

Affiliations:

[1]Neurodegeneration Pathobiology Laboratory, Queensland Brain Institute, University of Queensland, St Lucia, QLD, Australia

[2]Centre for Motor Neuron Disease Research, Macquarie Medical School, Macquarie University, Sydney, NSW, Australia

[3]Ionis Pharmaceuticals, Carlsbad, CA 90201, United States of America

***Corresponding author:**

Dr Adam K. Walker

E-mail: adam.walker@uq.edu.au

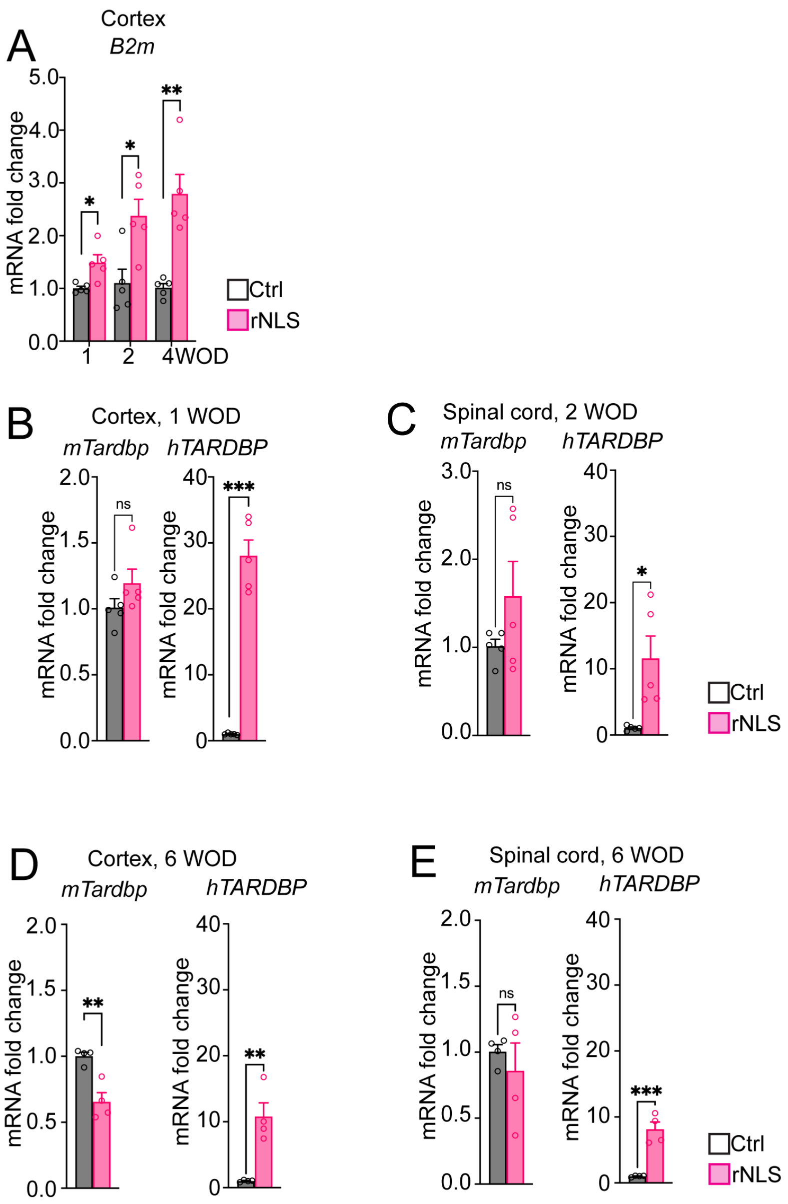

<u>Supplementary Fig. 1. Real-time qPCR analysis for *B2m*, mouse *Tdardbp* and human *TARDBP* genes.</u> **A**. Real-time qPCR data for the *B2m* gene in the cortex of C56BL/6JAusb mice at 1, 2 or 4 weeks off Dox (WOD). n = 5. Real-time qPCR data for mouse (m) *Tardbp* and human (h) *TARDBP* genes in the cortex of C56BL/6JAusb mice at 1 WOD (**B**), and the spinal cord at 2 WOD (**C**). n =5. Real-time qPCR data for *mTardbp* and *hTARDBP* genes in the cortex (**D**) and the spinal cord (**E**) of rNLS mice at 6 WOD. n = 4 . *Actb* was used as the housekeeping gene for normalisation. Mean <u>+</u> SEM. ns not significant, * $p < 0.05$, ** $p < 0.01$, and *** $p < 0.001$.

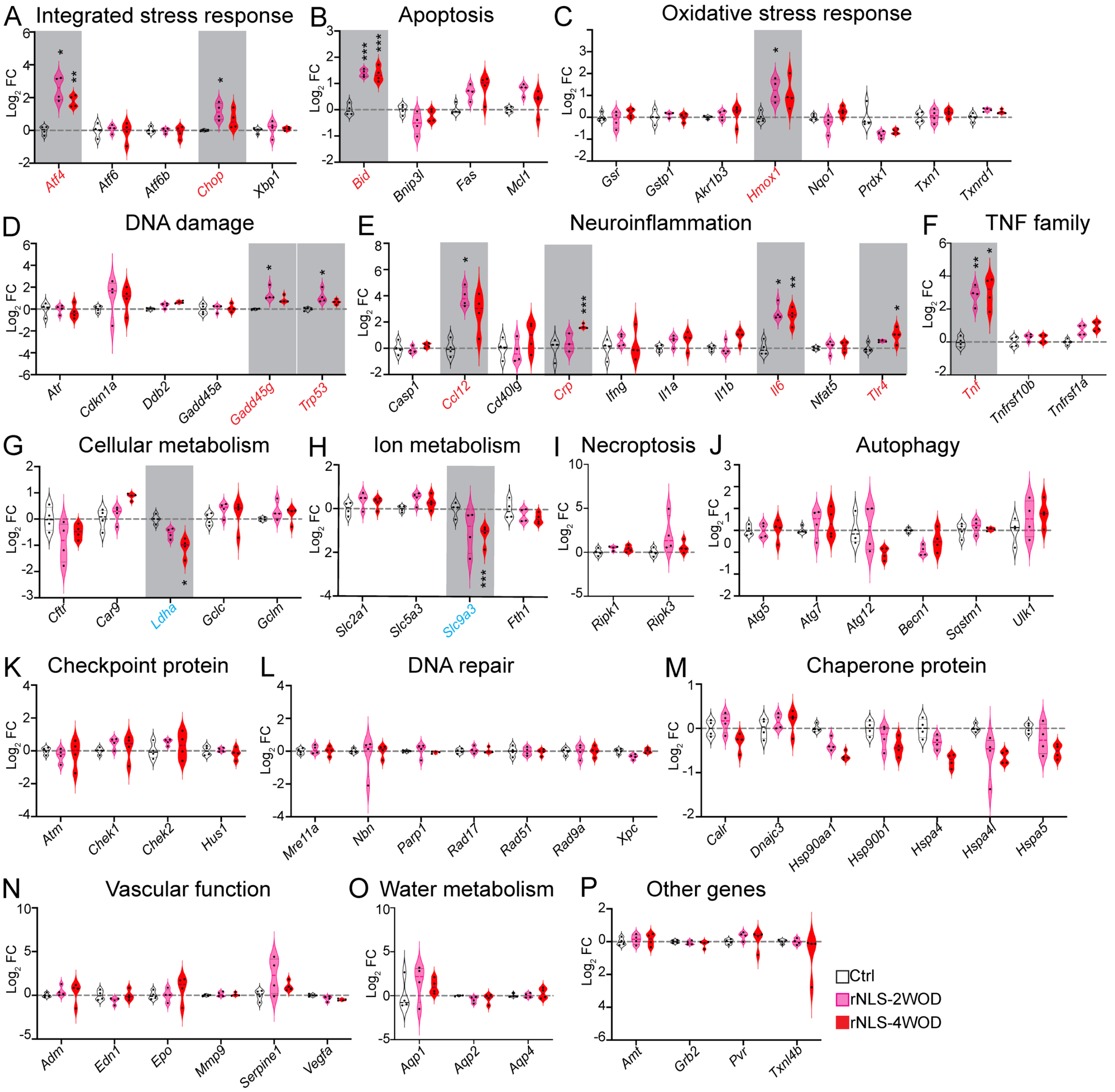

Supplementary Fig. 2. rNLS8 mice display significant dysregulation of genes in multiple cellular stress pathways in the cortex at early disease stages. The expression profiles for individual assayed genes from RT^2 PCR array analyses assigned to different biological functional groups at 2 and 4 weeks off Dox (WOD). **A**. Integrated stress response pathway. **B**. Apoptosis signaling. **C**. Oxidative stress response. **D**. DNA damage response. **E**. Neuroinflammation signaling. **F**. TNF family signaling. **G**. Cellular metabolism. **H**. Ion metabolism. **I**. Necroptosis. **J**. Autophagy. **K**. Checkpoint protein. **L**. DNA repair. **M**. Chaperone protein. **N**. Vascular function. **O**. Water metabolism. **P**. Remaining other genes. The statistically upregulated genes

(labels in red) and the downregulated genes (in blue) are highlighted in grey boxes. n = 4. ns not significant, * $p < 0.05$, ** $p < 0.01$ and *** $p < 0.001$.

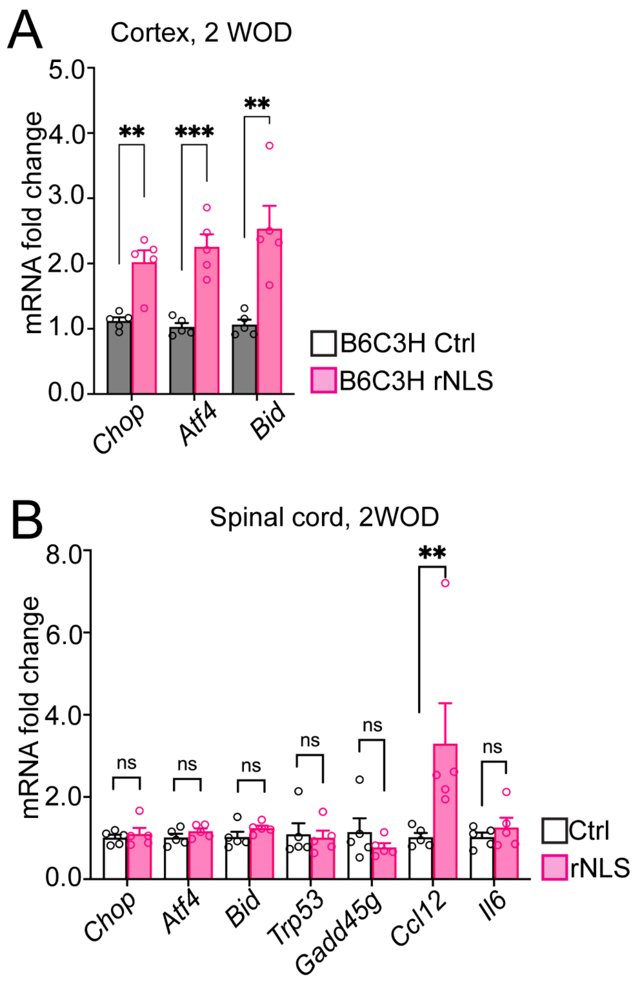

<u>Supplementary Fig. 3. Real-time qPCR data for representative genes identified in RT2 PCR array.</u> A. Real-time qPCR data for three RT^2 array representative genes in the cortex of B6C3H background mice at 2WOD. **B**. Representative genes in the lumbar spinal cord of C56BL/6JAusb mice at 2 WOD. n = 5. *Actb* was used as the housekeeping gene for normalisation. Mean <u>+</u> SEM. ns not significant, ** $p < 0.01$, and *** $p < 0.001$.

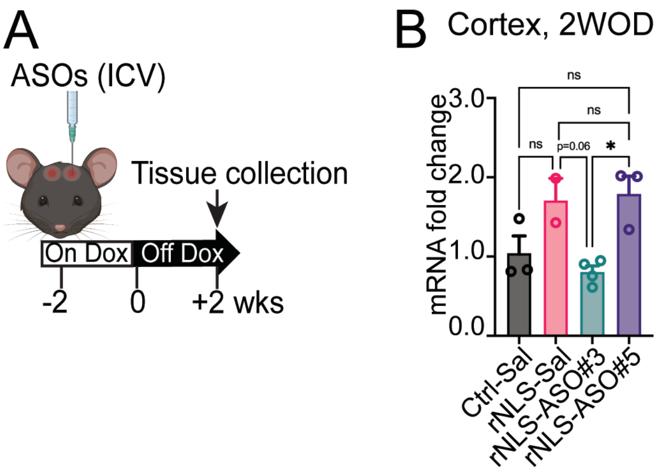

<u>Supplementary Fig. 4. *Chop* ASO#3 inhibits *Chop* gene expression in rNLS8 mice.</u> **A**. Experimental schema. **B**. Real-time qPCR results demonstrate knockdown of *Chop* gene by ASO #3 in the cortex of treated rNLS8 mice, but not by ASO #5. Saline-treated control mice (n = 3), saline-treated rNLS8 mice (n = 2), ASO#3-treated rNLS8 mice (n = 4) and ASO#5-treated rNLS8 mice (n = 3). *Actb* was used as the housekeeping gene for normalisation. Sequences of ASOs are available in Table 2. Mean $\pm$ SEM. ns not significant, * $p < 0.05$ (one-way ANOVA with Bonferroni's post hoc test).

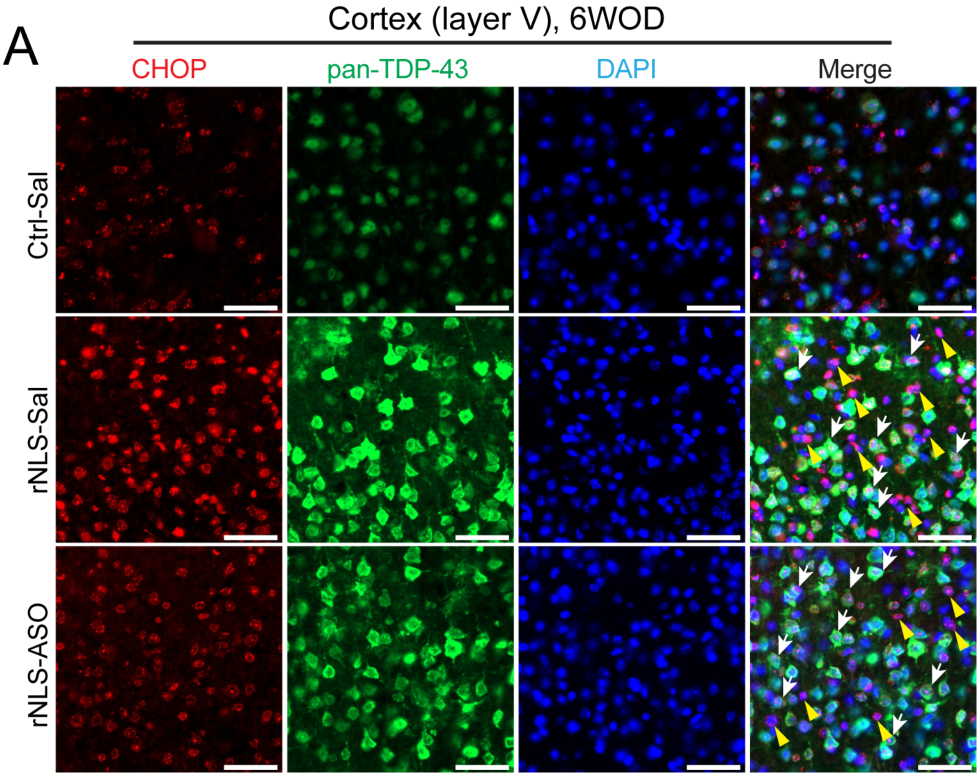

Supplementary Fig. 5. Representative IF images for CHOP (red), pan-TDP-43 (human + mouse) (green), the nuclear marker DAPI (blue), and the merged image in the layer V of cortex of Chop-ASO treated mice at 6 weeks off Dox (WOD). White arrows indicate CHOP and TDP-43 co-labelled neurons. Yellow solid triangles indicate CHOP positively labeled surrounding non-neuronal cells (TDP-43 negatively labelled). Scale bars represent 50 µm.

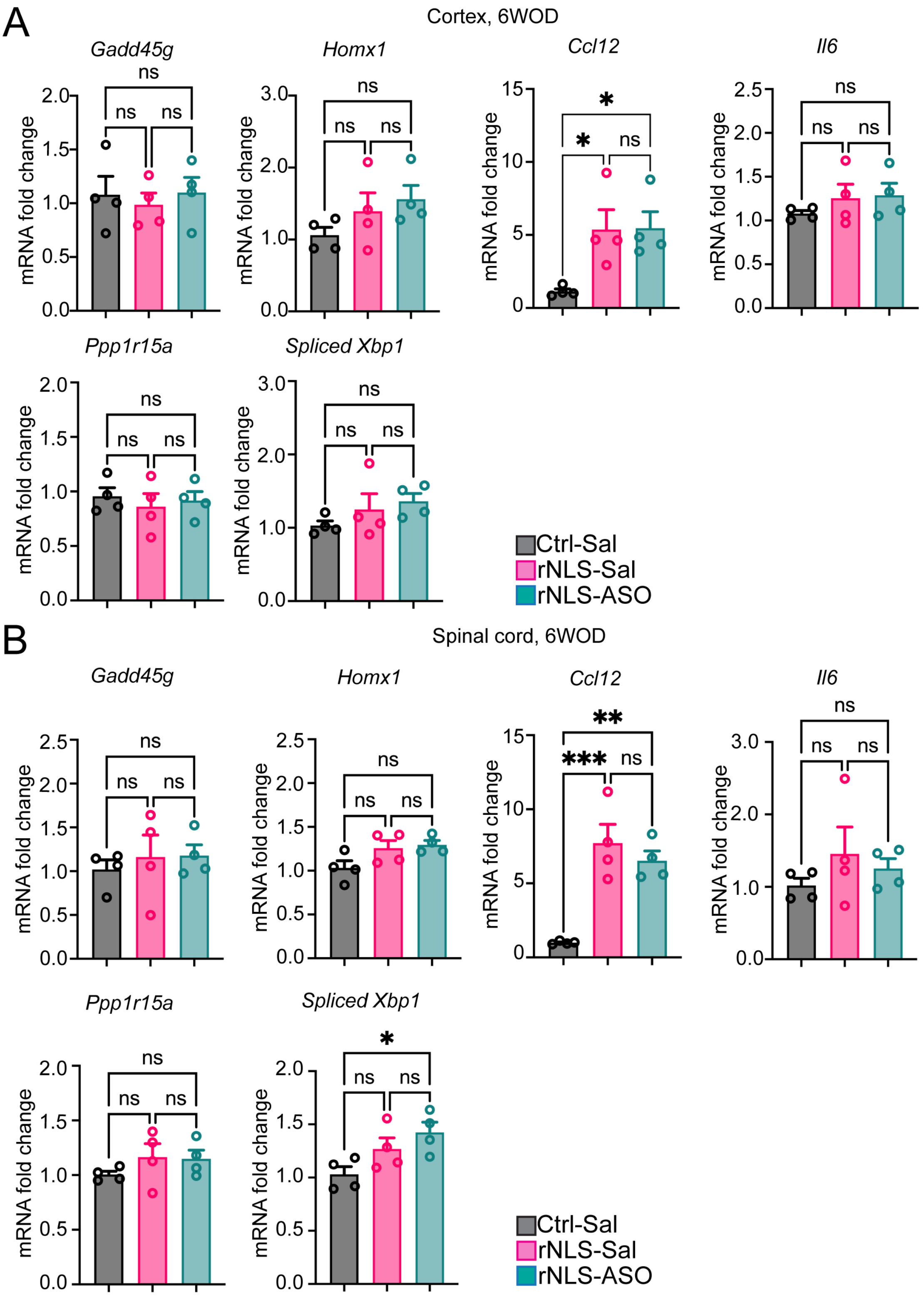

<u>Supplementary Fig. 6. *Chop* ASO does not change the expression of representative genes identified in RT^2 PCR array in rNLS8 mice.</u> Real-time qPCR analysis for array-identified representative genes in the cortex (**A**) and the spinal cord

(**B**) of *Chop* ASO-treated mice at 6 weeks off Dox (WOD). n = 4. Mean $\pm$ SEM. ns as not significant, * $p < 0.05$, ** $p < 0.01$, and *** $p < 0.001$.
